# Supplementary material for: Crossover behavior of the thermal conductance and Kramers’ transition rate theory
Source: Sci Rep. 2015 Dec 4;5:17506. doi: 10.1038/srep17506 (PMC4669443; doi:10.1038/srep17506)
Supplement: Supplementary Information [file srep17506-s1.pdf]

Supplemental information for "Crossover behavior of the thermal conductance and Kramers' transition rate theory"

Kirill A. Velizhanin,[1,*] Subin Sahu,[2] Chih-Chun Chien,[3] Yonatan Dubi,[4] and Michael Zwolak[2,5,†]

[1] *Theoretical Division, Los Alamos National Laboratory, Los Alamos, NM 87545, USA*

[2] *Department of Physics, Oregon State University, Corvallis, OR 97331, USA*

[3] *School of Natural Sciences, University of California, Merced, CA 95343, USA*

[4] *Department of Chemistry and the Ilse Katz Institute for Nanoscale Science and Technology, Ben-Gurion University of the Negev, Beer-Sheva 84105, Israel*

[5] *Center for Nanoscale Science and Technology, National Institute of Standards and Technology, Gaithersburg, MD 20899, USA*

I. DIMENSIONAL ANALYSIS OF THE UNIFORM HARMONIC MODEL

The complete list of parameters of the uniform harmonic model introduced in main article is: m, D, K, γ, $k_B T_L$, $k_B T_R$, N, N_r. We take m, K and $k_B \Delta T = k_B(T_L - T_R)$ as fundamental units to make all the other parameters dimensionless. Then, the independent dimensionless parameters of the model are

$$\frac{D}{K},\ \frac{\gamma}{\sqrt{mK}},\ \frac{T_L + T_R}{\Delta T},\ N \text{ and } N_r. \tag{1}$$

Using these dimensionless parameters, the *exact* expression for the heat current through the lattice can be written as

$$\frac{J}{k_B \Delta T}\sqrt{\frac{m}{K}} = C\left(\frac{D}{K}, \frac{\gamma}{\sqrt{mK}}, \frac{T_L + T_R}{\Delta T}, N, N_r\right), \tag{2}$$

where C is a dimensionless function of five dimensionless arguments. This very general expression can be simplified by (i) taking $N \to \infty$ (see inset in Fig. 2 of main article) and by (ii) exploiting the fact that the heat current in such a model is proportional to ΔT [see Eq. (17) and the related discussion], then the function C cannot depend on T_L and T_R. These considerations result in

$$\frac{J}{k_B \Delta T}\sqrt{\frac{m}{K}} = C\left(\frac{D}{K}, \frac{\gamma}{\sqrt{mK}}, N_r\right), \tag{3}$$

where C is now a dimensionless function of just three dimensionless arguments. The expression for heat conductance of the 1D uniform harmonic model considered in this work can then be written in the limit of $N \to \infty$ as

$$\sigma = k_B \sqrt{\frac{K}{m}} C\left(\frac{D}{K}, \frac{\gamma}{\sqrt{mK}}, N_r\right). \tag{4}$$

* kirill@lanl.gov

† mpz@nist.gov

II. NUMERICAL SOLUTION OF LANGEVIN EQUATIONS OF MOTION

The Langevin equations of motion for a lattice of harmonic oscillators, given by the Hamiltonian

$$H = \frac{1}{2}\mathbf{p}^{\intercal}\mathbf{M}^{-1}\mathbf{p} + \frac{1}{2}\mathbf{x}^{\intercal}\mathbf{K}\mathbf{x}, \tag{5}$$

are a system of first-order linear differential equations

$$\begin{aligned} \dot{x}_n &= p_n/m_n, \\ \dot{p}_n + \sum_{m=1}^{N} K_{nm}x_m + \gamma_n p_n/m_n &= \eta_n(t), \end{aligned} \tag{6}$$

where the mass can potentially be site-dependent in order to, e.g., examine mass disorder as in Disordered Harmonic Lattice discussed in main article. The matrix $\mathbf{K}$ contains the interaction potentials and $[\mathbf{M}]_{nm} = \delta_{nm}m_n$ is a diagonal matrix of the masses. These equations of motion can be expressed as

$$\frac{d}{dt}|q(t)\rangle + \mathbf{G}|q(t)\rangle = |\eta(t)\rangle, \tag{7}$$

where

$$|q\rangle \Rightarrow \begin{pmatrix} \vec{x} \\ \vec{p} \end{pmatrix}. \tag{8}$$

The non-symmetric matrix $\mathbf{G}$ is given by

$$\mathbf{G} = \begin{pmatrix} \mathbf{0} & -\mathbf{M}^{-1} \\ \mathbf{K} & \mathbf{\Gamma}\mathbf{M}^{-1} \end{pmatrix}, \tag{9}$$

where $\mathbf{\Gamma}$ is a diagonal matrix of the couplings to the Langevin reservoirs (and $\mathrm{tr}|\mathbf{\Gamma}| = 2\Gamma$, where Γ is the cumulative friction coefficient).

The formal solution of Eq. (7) is

$$|q(t)\rangle = \int_{t_0}^{t} dt'\, e^{-\mathbf{G}(t-t')}|\eta(t')\rangle. \tag{10}$$

An important class of observables can be expressed as an average of a quadratic form of $|q(t)\rangle$ over the statistical ensemble. For example, the heat current flowing from site n to site $n+1$ can be written as a statistical average of

$$J_n = -\frac{K_{n+1,n}}{m_{n+1}} p_{n+1}x_n. \tag{11}$$

An observable of this class can be represented by an operator $\mathbf{O}$ so that

$$O(t) = \langle q(t)|\mathbf{O}|q(t)\rangle$$
$$= \int_{t_0}^{t} dt' \int_{t_0}^{t} dt'' \, \langle \eta(t')|e^{-\mathbf{G}^\dagger (t-t')}\mathbf{O}e^{-\mathbf{G}(t-t'')}|\eta(t'')\rangle. \tag{12}$$

Averaging over the statistical ensemble and using Eq. 15 from Methods section of main article for the auto-correlation function of the random forces, one obtains

$$O(t) = \int_{t_0}^{t} dt' \, \mathrm{Tr}\left[e^{-\mathbf{G}^\dagger (t-t')}\mathbf{O}e^{-\mathbf{G}(t-t')}\mathbf{F}\right], \tag{13}$$

where $\mathbf{F}$ is a diagonal matrix with its only non-zero elements given by

$$F_{N+n,N+n} = 2\gamma_n k_B T_n, \;\; n = 1, 2, ..., N. \tag{14}$$

The spectral decomposition of the particular class of non-symmetric operators $\mathbf{G}$ above necessarily includes left and right eigenvectors, i.e., $\mathbf{G} = \sum_k \lambda_k |k_R\rangle\langle k_L|$, where the components of right (column) and left (row) eigenvectors are denoted by $[|k_R\rangle]_n = v^R_{n,k}$ and $[\langle k_L|]_n = \left(v^L_{n,k}\right)^*$, respectively. The orthonormality condition is $\langle k_L|l_R\rangle = \sum_{n=1}^{2N} \left(v^L_{n,k}\right)^* v^R_{n,l} = \delta_{kl}$. The spectral decomposition of $\mathbf{G}^\dagger$ is given by $\mathbf{G}^\dagger = \sum_k \lambda_k^* |k_L\rangle\langle k_R|$, where $|k_L\rangle = \langle k_L|^\dagger$ and $\langle k_R| = |k_R\rangle^\dagger$.

Substituting these spectral decompositions into Eq. (13), setting $t_0 \to -\infty$ to find the steady-state value of the observable, and integrating, we obtain

$$O = \sum_{k,l=1}^{2N} \frac{\langle l_R|\mathbf{O}|k_R\rangle\langle k_L|\mathbf{F}|l_L\rangle}{\lambda_k + \lambda_l^*}. \tag{15}$$

This equation allows one to draw very general conclusions regarding the dependence of the observable O on temperature without the need to explicitly specify $\mathbf{G}$ and $\mathbf{O}$. Since operators $\mathbf{O}$ (e.g., heat current) and $\mathbf{G}$ do not typically depend on temperature, and the operator $\mathbf{F}$ in Eq. (14) is strictly linear with respect to temperature, O is then a linear form of $T_1, T_2, ..., T_N$. Specifically, since in the harmonic lattice case we examine there is only two independent temperatures, T_L and T_R, O is necessary a linear form of T_L and T_R, i.e.,

$$O(T_L, T_R) = \alpha_L T_L + \alpha_R T_R, \tag{16}$$

where α_L and α_R are coefficients that depend only on specific parameters of the system and nature of operator $\mathbf{O}$, but not temperature. For example, since the heat current has to vanish at thermodynamic equilibrium, we must have $\alpha_L = -\alpha_R$ and, therefore,

$$J \propto T_L - T_R. \tag{17}$$

That is, the heat conductance does not depend on temperatures T_L or T_R at all. These considerations do not lead to

the same conclusion in the case of anharmonic lattices, as illustrated in Fig. 5(b).

III. HEAT CURRENT IN 1D HARMONIC LATTICES

To use Eq. (15) to evaluate the heat current in the lattice of harmonic oscillators, one needs to construct operators $\mathbf{G}$ and $\mathbf{O}$. Below we explicitly construct these matrices and derive an expression for the heat current.

The system under consideration is a lattice of harmonic oscillators with only nearest neighbor coupling, which yields a matrix $\mathbf{K}$ in Eq. (5) that is sparse. For an oscillator with index n somewhere inside the lattice (i.e., $1 < n < N$), the corresponding n^{th} row of the matrix has only three non-zero components $(K_{n,n-1}, K_{n,n}, K_{n,n+1}) = (-K, D{+}2K, -K)$, where K and D are parameters in the Hamiltonian. The end sites, $n = 1$ and $n = N$, are assumed to be coupled to "hard walls", i.e., to implicit oscillators with indices $n = 0$ and $n = N + 1$, respectively, whose coordinates are kept zero. This results in only two non-zero matrix elements in the very first and the very last rows of matrix $\mathbf{K}$: $(K_{1,1}K_{1,2}) = (D + 2K, -K)$ and $(K_{N,N-1}K_{N,N}) = (-K, D + 2K)$.

According to Eq. (11), the operator $\mathbf{J}_n$ corresponding to the heat current from site n to site $n+1$ has only a single non-zero element

$$[\mathbf{J}_n]_{N+n+1,n} = \frac{K}{m_{n+1}}. \tag{18}$$

Substituting this expression, together with Eq. (14) and the eigenvectors of $\mathbf{G}$ and $\mathbf{G}^\dagger$, into Eq. (15) gives

$$J_n = \frac{2k_B K}{m_{n+1}} \sum_{k,l=1}^{2N} \left[\frac{\left(v^R_{N+n+1,l}\right)^* v^R_{n,k}}{\lambda_k + \lambda_l^*} \times \sum_{m=1}^{N} \gamma_m T_m \left(v^L_{N+m,k}\right)^* v^L_{N+m,l} \right]. \tag{19}$$

IV. HEAT CURRENT IN THE SMALL γ REGIME

The most convenient way to analyze the small γ dynamics of the 1D lattice is to perform a perturbation expansion of eigenvalues and eigenvectors of operator $\mathbf{G}$ with respect to γ. $\mathbf{G}$ is at most linear with respect to γ and can be represented as $\mathbf{G} = \mathbf{G}_0 + \gamma\mathbf{G}_1$, where $\mathbf{G}_{0,1}$ are independent of γ. The zeroth-order eigenvalues and eigenvectors of $\mathbf{G}$ are then the eigenvalues and eigenvectors of $\mathbf{G}_0$,

$$\mathbf{G}_0 = \begin{pmatrix} \mathbf{0} & -\mathbf{M}^{-1} \\ \mathbf{K} & \mathbf{0} \end{pmatrix}. \tag{20}$$

The solutions – normal modes of lattice vibrations with no friction – can be found in the standard way, first by scaling ("mass-weighting") by $\mathbf{S} = \mathrm{diag}(\mathbf{M}^{1/2}, \mathbf{M}^{-1/2})$,

$$\mathbf{S}\mathbf{G}_0\mathbf{S}^{-1} = \begin{pmatrix} \mathbf{0} & -\mathbf{I} \\ \mathbf{M}^{-\frac{1}{2}}\mathbf{K}\mathbf{M}^{-1/2} & \mathbf{0} \end{pmatrix} \tag{21}$$

and then diagonalizing the mass-weighted coupling $\mathbf{M}^{-1/2}\mathbf{K}\mathbf{M}^{-1/2}$ via an orthogonal transformation $\mathbf{T}$. The normal modes of the lattice are

$$x_n^k(t) = u_n^k e^{\pm i\omega_k t}, \tag{22}$$

where $u_n^k = \left[\mathbf{M}^{-1/2}\mathbf{T}\right]_{nk}$ are the real-valued polarization vectors of the normal modes and $n, k = 1, \ldots, N$ enumerate the lattice sites and modes, respectively.

The eigenvalues and *right* eigenvectors (hence denoted with R below) of $\mathbf{G}_0$ can be readily constructed from these and they come in pairs. The unnormalized "positive frequency" solutions are

$$\begin{aligned} \lambda_k^0 &= i\omega_k, \\ v_{n,k}^{0,R} &\propto u_n^k, \\ v_{N+n,k}^{0,R} &\propto -i\omega_k m_n u_n^k, \end{aligned} \tag{23}$$

for $k = 1, \ldots, N$ and $n = 1, \ldots, N$. The unnormalized "negative frequency" solutions are

$$\begin{aligned} \lambda_k^0 &= -i\omega_k, \\ v_{n,k}^{0,R} &\propto u_n^k, \\ v_{N+n,k}^{0,R} &\propto i\omega_k m_n u_n^k, \end{aligned} \tag{24}$$

for $k = N+1, \ldots, 2N$, $n = 1, \ldots, N$, and "extending" $\omega_k = \omega_{k-N}$ and $u_n^k = u_n^{k-N}$ for $k > N$. We have ordered the solution so that when the positive frequency solution takes on the label k, the negative frequency solution takes on the label $k + N$. The last two lines of each of these solutions reflect the simple relationship between momenta and the time derivative of coordinates, the first line in Eq. (6).

The left eigenvectors can be found in a similar way, essentially the position and momenta change roles including how they are scaled by mass. With the convention that $[\langle k_L||]_n = \left(v_{n,k}^L\right)^*$, the unnormalized "positive frequency" solutions are

$$\begin{aligned} \lambda_k^0 &= i\omega_k, \\ v_{n,k}^{0,L} &\propto i\omega_k m_n u_n^k, \\ v_{N+n,k}^{0,L} &\propto u_n^k, \end{aligned} \tag{25}$$

for $k = 1, \ldots, N$ and $n = 1, \ldots, N$. The unnormalized "negative frequency" solutions are

$$\begin{aligned} \lambda_k^0 &= -i\omega_k, \\ v_{n,k}^{0,L} &\propto -i\omega_k m_n u_n^k, \\ v_{N+n,k}^{0,L} &\propto u_n^k, \end{aligned} \tag{26}$$

for $k = N+1, \ldots, 2N$, $n = 1, \ldots, N$, and the same "extension" for $k > N$. The left and right eigenvectors clearly

obey the right orthogonality relationship, but for each k have norm $\mp 2i\omega_k$ for "positive" and "negative" frequencies, respectively.

The first-order perturbative correction to the eigenvalues of $\mathbf{G}_0$ can be found in the usual manner, i.e.,

$$\lambda_k^1 = \gamma \langle k_L^0 | \mathbf{G}_1 | k_R^0 \rangle. \tag{27}$$

Using this expression, we can now expand the current. To do so, we will make use of Eq. (15). The matrix element $\langle k_L | \mathbf{F} | l_L \rangle$ is linear with respect to γ at small γ, i.e., putting in the normalized zeroth order states $\langle k_L^0 | \mathbf{F} | l_L^0 \rangle = \sum_n 2\gamma_n k_B T_n u_n^k u_n^l / \sqrt{4\omega_k \omega_l}$. Thus, we can focus on the quantity

$$\frac{\langle l_R | \mathbf{J}_n | k_R \rangle}{\lambda_k + \lambda_l^*} \tag{28}$$

and show that it is zeroth order with respect to γ. We separate the summation in Eq. (15) into the off-diagonal ($k \neq l$) and diagonal ($k = l$) contributions. Assuming no degeneracy, the former is nonzero and well-behaved when zeroth-order eigenvalues and eigenvectors are used since the denominator in Eq. (15) does not vanish at $k \neq l$. The off-diagonal contribution is thus proportional to γ at small γ.

The diagonal contribution has a vanishing denominator as γ approaches zero, i.e., λ_k^0 is pure imaginary which gives $\lambda_k + \lambda_k^* = 0 + \sum_n \gamma_n (u_n^k)^2 + \mathcal{O}(\gamma^2)$. Performing the whole sum, however, we can pair the "positive" and "negative" frequencies:

$$\sum_{k=1}^{N} \left(\langle k_R | \mathbf{J}_n | k_R \rangle + \langle (k+N)_R | \mathbf{J}_n | (k+N)_R \rangle \right) \frac{\langle k_L^0 | \mathbf{F} | k_L^0 \rangle}{\lambda_k + \lambda_k^*} \tag{29}$$

where the last factor is the same for both "positive" and "negative" frequencies. The term in parenthesis gives

$$\left(\frac{i m_{n+1} u_{n+1}^k u_n^k}{2} + \frac{-i m_{n+1} u_{n+1}^k u_n^k}{2} + \mathcal{O}(\gamma) \right) = 0 + \mathcal{O}(\gamma) \tag{30}$$

Thus, the sum over "positive" and "negative" frequencies gives a contribution that is of order γ. Therefore, the current for small γ is $J \propto \gamma$.

V. HEAT CURRENT IN THE LARGE γ REGIME

The current in the large γ regime can also be calculated perturbatively in powers of $1/\gamma$. We consider only single sites at each end connected to Langevin reservoirs, as any additional sites in the extended reservoir are decoupled from the lattice by higher orders in $1/\gamma$. Now consider the matrix $\gamma \mathbf{G}_1 + \mathbf{G}_0$, where $\mathbf{G}_0$ is the perturbation. The expansion is complicated by the fact that the "bare" matrix

$$\gamma \mathbf{G}_1 = \begin{pmatrix} \mathbf{0} & \mathbf{0} \\ \mathbf{0} & \mathbf{\Gamma} \mathbf{M}^{-1} \end{pmatrix} \tag{31}$$

$\downarrow k \neq l \rightarrow$	$l \in \mathcal{B}_{-1}$	$l \in \mathcal{B}_0$	$l \in \mathcal{B}_1$
$k \in \mathcal{B}_{-1}$	$\frac{1}{\gamma}; \frac{1}{\gamma}; \frac{1}{\gamma}$	$1; \frac{1}{\gamma}; 1$	$\frac{1}{\gamma}; 1; \gamma$
$k \in \mathcal{B}_0$	$1; \frac{1}{\gamma}; 1$	$1; \frac{1}{\gamma}; 1$	$1; 1; \gamma$
$k \in \mathcal{B}_1$	$\frac{1}{\gamma}; 1; \gamma$	$1; 1; \gamma$	$\frac{1}{\gamma}; 0; \gamma$

TABLE I. Order of the different factors in the current contribution, Eq. 15, when $k \neq l$. Note that cross terms between $\mathcal{B}_1$ and $\mathcal{B}_1$ are identically zero given our choice of zeroth order eigenvectors.

is highly degenerate, with $2N-2$ zero eigenvalues and 2 non-zero eigenvalues. The latter two are γ/m_1 and γ/m_N, which may have an (unimportant) degeneracy, and we can take two of the zeroth order right (left) eigenvectors to be $|N+1\rangle$ and $|2N\rangle$ ($\langle N+1|$ and $\langle 2N|$).

The degenerate space is spanned by the states $|n\rangle$ with $n = 1, \ldots, N$ and $|N+m\rangle$ with $m = 2, \ldots, (N-1)$. The degeneracy is lifted in the normal way: Let $\mathbf{P}_0$ project onto this subspace and diagonalize $\mathbf{P}_0\mathbf{G}_0\mathbf{P}_0$. This matrix has $2N-4$ eigenvectors with non-zero eigenvalues. The right eigenvectors with non-zero eigenvalues have the same form as Eqs. (23) and (24) except found from the lattice sites $2, \ldots, (N-1)$ only. The other two eigenvectors have a zero eigenvalue. Their degeneracy is not broken until the next order ($1/\gamma$), and requires diagonalizing

$$\mathcal{G} = \mathbf{P}_0\mathbf{G}_0\mathbf{P}_0 - \frac{1}{\gamma}\mathbf{P}_0\mathbf{G}_0\tilde{\mathbf{P}}_0\frac{1}{\mathbf{G}_1}\tilde{\mathbf{P}}_0\mathbf{G}_0\mathbf{P}_0, \tag{32}$$

with $\tilde{\mathbf{P}}_0 = (\mathbf{I} - \mathbf{P}_0)$. This matrix has a simple form

$$\begin{aligned}\mathcal{G} = &-\sum_{n\neq 1,N}\frac{1}{m_n}|n\rangle\langle N+n| + \sum_{n\neq 1,N;m} K_{nm}|N+n\rangle\langle m| \\ &+\frac{1}{\gamma}\sum_{n=1,N;m} K_{nm}|n\rangle\langle m|.\end{aligned} \tag{33}$$

Using this, we can search for the remaining two linearly independent and stable right eigenvectors with an eigenvalue of order $1/\gamma$. This results in a generalized eigenvalue problem for the zeroth order right eigenvectors, $\mathbf{K}|\lambda^0\rangle = \lambda\mathbf{B}|\lambda^0\rangle$ where $[\mathbf{B}]_{nm} = \delta_{nm}(\delta_{n1} + \delta_{nN})$. When this eigenvector is extended to the full degenerate space (padding it with zeros for all the momentum components), one has $\mathcal{G}|\lambda^0\rangle \approx \lambda/\gamma|\lambda^0\rangle$.

Starting with these zeroth order eigenvectors, then we can apply perturbation theory as usual. This results in three sets of eigenvectors, ($\mathcal{B}_1$) ones with eigenvalues that are $\mathcal{O}(\gamma)$ ($|N+1\rangle$ and $|2N\rangle$), ($\mathcal{B}_0$) ones that are $\mathcal{O}(1)$ (Eqs. (23) and (24) but in the internal lattice), and ($\mathcal{B}_{-1}$) ones that are $\mathcal{O}(1/\gamma)$ ($\mathbf{K}|\lambda^0\rangle = \lambda\mathbf{B}|\lambda^0\rangle$). We can also delineate these sets by the subspace on which the zeroth order eigenvectors live ($\mathcal{B}_1$ on ($|N+1\rangle$ and $|2N\rangle$; $\mathcal{B}_0$ on $|n\rangle$ with $n = 2, \ldots, (N-1)$ and $|N+m\rangle$ with $m = 2, \ldots, (N-1)$; $\mathcal{B}_{-1}$ on $|n\rangle$ with $n = 1, \ldots, N$). Contributions to the eigenvectors outside this subspace will be order $1/\gamma$. As with the small γ regime, we need to group the different contributions into diagonal and off-diagonal (and then further distinguish between off-diagonal contributions between these three groups). We will break down the contributions to Eq. (15) in terms of each of the factors $\langle l_R|\mathbf{J}|k_R\rangle$, $\langle k_L|\mathbf{F}|l_L\rangle$, and $\lambda_k + \lambda_l^*$.

We start with the diagonal contributions. For eigenvectors in $\mathcal{B}_1$, we get contributions to $\langle l_R|\mathbf{J}|k_R\rangle$; $\langle k_L|\mathbf{F}|l_L\rangle$; $\lambda_k+\lambda_l^*$ of: at most $\mathcal{O}(1/\gamma)$ due to the zeroth order states living on $\{|N+1\rangle, |2N\rangle\}$; $\mathcal{O}(\gamma)$ as, e.g., $\langle N+1|\mathbf{F}|N+1\rangle = \mathcal{O}(\gamma)$; $\mathcal{O}(\gamma)$ because $\lambda_k \approx \gamma$. This gives an overall contribution at most of order $1/\gamma$.

For eigenvectors in $\mathcal{B}_0$, we get: $\mathcal{O}(1)$ (this is the same calculation as Eq. (30) as the states are eigenstates of $\mathbf{G}_0$ on the internal lattice); $\mathcal{O}(1/\gamma)$ because although $\mathbf{F}$ is order γ, the contribution of $|N+1\rangle$ and $|2N\rangle$ to the eigenvector are order $1/\gamma$; $\mathcal{O}(1/\gamma)$ because the eigenvalue has a real part that is order $1/\gamma$ (the imaginary part is order 1 but this cancels in $\lambda_k+\lambda_l^*$). Thus, the total contribution from a eigenvector in $\mathcal{B}_0$ is order 1. However, just like in Eq. (30) for the small γ regime, this contribution always has a paired contribution from the negative (positive) frequency mode, which cancels this order 1 contribution. This gives an overall contribution at most of order $1/\gamma$.

For eigenvectors in $\mathcal{B}_{-1}$, we get: $\mathcal{O}(1/\gamma)$ due to the zeroth order eigenvector living only on the position component of the vector; $\mathcal{O}(1/\gamma)$ because although $\mathbf{F}$ is order γ, the contribution of $|N+1\rangle$ and $|2N\rangle$ to the eigenvector are order $1/\gamma$; $\mathcal{O}(1/\gamma)$ because the eigenvalue has a real part of order $1/\gamma$. This gives an overall contribution at most of order $1/\gamma$.

For the off-diagonal terms, we give the contributions to each of the three factors for all the different possibilities in Table I. The highest order contributions from the off diagonal are order $1/\gamma$. Thus, the sum over all contributions gives zero for the zeroth order term and the leading term is order $1/\gamma$.

We note that for both the small γ and large γ regimes, these well-defined perturbation expansions demonstrate that the the zeroth order terms are zero, a fact which is obvious for the small γ regime (i.e., there would be no driving force for the thermal current) but not the large γ regime. Moreover, due to the presence of the different temperature reservoirs at each end, the first order expressions are non-zero always unless the lattice has a broken link. Coupled with the results of the anharmonic lattice, the perturbative expressions give evidence that the presence of these regimes are universal except in pathological cases.
